# Differential Patterns of Food Appreciation during Consumption of a Simple Food in Congenitally Anosmic Individuals: An Explorative Study

**DOI:** 10.1371/journal.pone.0033921

**Published:** 2012-04-18

**Authors:** Lenka Novakova, Viola Bojanowski, Jan Havlíček, Ilona Croy

**Affiliations:** 1 Department of Anthropology, Faculty of Humanities, Charles University, Prague, Czech Republic; 2 Interdisciplinary Center for Smell and Taste Research, Department of Otorhinolaryngology, University of Dresden Medical School, Dresden, Germany; Duke University, United States of America

## Abstract

Food is evaluated for various attributes. One of the key food evaluation domains is hedonicity. As food is consumed, its hedonic valence decreases (due to prolonged sensory stimulation) and hedonic habituation results. The aim of the present study was to investigate changes in food pleasantness ratings during consumption of a simple food by individuals without olfactory experience with food as compared to normosmics. 15 congenital anosmics and 15 normosmic controls were each presented with ten 10 g banana slices. Each was visually inspected, then smelled and chewed for ten seconds and subsequently rated for hedonicity on a 21-point scale. There was a significant difference in pleasantness ratings between congenital anosmics and controls (F(1, 26) = 6.71, p = .02) with the anosmics exhibiting higher ratings than the controls, a significant main repeated-measures effect on the ratings (F(1.85, 48) = 12.15, p<.001), which showed a decreasing trend over the course of consumption, as well as a significant portion*group interaction (F(1.85, 48) = 3.54, p = .04), with the anosmic participants experiencing a less pronounced decline. The results of the present explorative study suggest that over the course of consumption of a simple food, congenitally anosmic individuals experience differential patterns of appreciation of food as compared to normosmics. In this particular case, the decrease of hedonic valence was less pronounced in congenital anosmics.

## Introduction

Food is evaluated for various attributes through several sensory modalities. The sensory perception of food involves vision, smell, taste, touch, audition and the trigeminal somatosensory system [1] as well as the sensory receptors in the digestive tract and circulatory system [2]. The food's location is identified at a distance using orthonasal olfaction, substantially facilitated by visual cues [3], which may, even at close proximity, override olfactory perception [4]. When the food is delivered to the mouth, but prior to ingestion, it is assessed on the basis of a multimodal sensory integration of retronasal olfaction, taste, and somatosensory input such as mechano-sensation, temperature or irritation [5].

One of the key domains of food evaluation is hedonicity. Over the longer term, it is thought that foods acquire hedonic valence mainly through various learning processes; a unique set of food likes and dislikes is formed over the life course based on the individual's experiences and socially held beliefs. Undoubtedly, one of the key guides in this process is food flavour, and the most widely cited learning models are those based on flavour-based learning, namely those proposing associations between a novel flavour and an existing liked or disliked flavour, or post-ingestive consequences, ingestion of nutrients in particular (for review, see [6]).

Over the short-term, positive hedonic evaluation (liking, pleasantness, appreciation) reflects the immediate experience or anticipation of pleasure from the orosensory stimulation of eating a food. This is referred to as *palatability* [7], and has a positive effect on food intake [8], known as the *appetizer effect* [9]. The driving force behind this effect is the food's flavour, so evidently the retronasal olfactory component comes into play here.

However, the pleasantness of a particular food varies over time. During a meal, the hedonic assessment of the food's visual, olfactory and gustatory properties typically decreases [10]. Accordingly, along with the decline of sensory-mediated pleasantness, the reward value of a particular food decreases during its consumption because of repeated exposure to a particular sensory signal, a phenomenon referred to as *sensory-specific satiation* [11]. In other words, repeated exposure to a food over the course of consumption results in what has been defined as “boredom with taste” [11].

*Sensory-specific satiation* is facilitated by exposure time [12]–[13], sensory complexity of the food [14], and intensity [15]. This is not to be confused with *sensory-specific satiety*, a phenomenon that refers to the decline in pleasantness of a particular food when compared to the pleasantness of uneaten foods [16]. Special cases would be the (partial) olfactory and taste sensory-specific satieties, which do not depend on the ingestion of nutrients [17].

Although there is uncertainty as to whether it is the sensory-specific satiation or the satiety phenomenon that bears the major responsibility for the drive for variety and food choice, this makes sense from an evolutionary viewpoint, since it increases the chance of having an adequate intake of various nutrients, and reduces the risk of a toxic overload from one food [18].

Being attracted to a food odor is not the sole driving force for food intake because people with olfactory and gustatory disorders still have a drive to eat and they do not necessarily consume less food than individuals with intact senses of smell and taste [19]–[20], (although a self-reported decrease of appetite in patients with olfactory dysfunction has been reported [21]). More frequently, people with olfactory loss have reported reduced food appreciation [21]–[24]. This is of little surprise as, despite normal gustatory function, anosmic individuals have an impaired appreciation of food flavor.

Patients have reported several ways of coping with various olfactory disorders. The most intriguing group of patients are those who have been diagnosed with congenital anosmia. They are of particular interest because of their lifelong lack of olfactory experience with food. Congenitally anosmic individuals tend to focus on the primary tastes, and seek foods with pleasant textures [25] and those which stimulate the trigeminal nerve [26].

Nevertheless, these (often isolated) self-reports, however valuable, do not provide us with an understanding of whether the appreciation of a simple food over the course of consumption is affected by congenital anosmia. This is of interest because the decline of hedonic valence seems to play a crucial role in sensory-specific satiation. We hypothesized that over the course of consumption of a simple, single-food snack-size meal, congenitally anosmic individuals would exhibit a different pattern of change in pleasantness ratings, compared to normosmic controls; namely, that the expected decrease would be delayed and less pronounced in congenital anosmics.

Thus, the aim of the present study was to track the changes in the pleasantness of a simple food over the course of a serving in congenital anosmics and compare them with the results from healthy control subjects.

## Materials and Methods

### Participants

Fifteen individuals with congenital anosmia (13 women, 2 men; mean [SD] age, 31.0 [9.9] years, range 20–42 years) and fifteen normosmic controls (12 women, 3 men; mean [SD] age, 27.8 [5.2] years, range 21–39 years) participated in the study. The recruitment of congenitally anosmic participants was carried out while another study was being conducted at the research centre. We invited the participation of congenitally anosmic individuals who were listed in the centre's long-term database and who were participating in a study concerning the effects of olfactory loss on taste perception and quality of life. Congenital anosmia was diagnosed using (1) detailed medical history, with participants mentioning no previous taste of flavor experience in their lives, (2) psychophysical examination using the Sniffin' Sticks, with TDI scores less than 15.5, indicative of functional anosmia, (3) electrophysiological measurements based on olfactory event-related potentials, whch were absent in all subjects, and (4) magnetic resonance imaging with severe hypoplasia or aplasia of the olfactory bulb and an olfactory depth of less than 8.0 mm in the plane of the posterior tangent through the eyeballs. The control participants' normal olfactory function was ascertained by use of the extended version of the ‘Sniffin’ Sticks' test. All of the participants were instructed to refrain from food two hours prior to the commencement of the study. The two groups did not differ in age (t_28_ = 1.01, p = .32) or age distribution (χ^2^ = 2.40, p = .12), socioeconomic status based on educational background (t_28_ = 1.83, p = .08), BMI (t_28_ = .75, p = .46), time lag between the last meal and their participation in the study (t_28_ = .27, p = .79), estimated calories consumed prior to participation (t_28_ = .66, p = .52) or self-assessed hunger (t_28_ = .40, p = .69), which was indicated on a 21-point scale, ranging from −10 and 10 (extremely hungry and not hungry at all, respectively).

### Ethics Statement

Investigations were performed in accordance with the Declaration of Helsinki on Biomedical Research Involving Human Subjects; every participant provided written informed consent. The research was approved by the IRB Charles University, Faculty of Sciences.

### Procedure

Before taking part in the study, each participant had already spent an average time of 90 minutes at the clinic, ensuring that no food was consumed immediately before the test began. Since most appointments were scheduled for late in the morning, the last meal reported in the vast majority of cases consisted of moderate amounts of wholemeal bakery products. Care was taken that the room in which the session was to take place was well ventilated and free of any possibly disturbing odors.

Immediately prior to the commencement of the session, ten fresh banana slices were prepared out of the participant's sight. Each portion weighed 10 grams. Banana was chosen as a stimulus due to its low trigeminal activation, soft texture, and the fact that its odor pleasantness is widely agreed upon [27]. In the meanwhile, the participant was seated and asked to fill in a brief questionnaire regarding their last meal in which they were to specify items and amounts consumed, the time elapsed since that last meal and their level of hunger. Subsequently, a plate with the banana slices was placed in front of the participant and a PowerPoint presentation (Microsoft Corporation, Redmond, WA, USA) was run to deliver instructions and to pace the session. To prolong the exposure time, each slice was to be consumed in the following manner: first, the participant was asked to take a slice of banana in the hand and inspect it visually for ten seconds. Next, it was to be smelled and then it was to be chewed without swallowing, each for a period of ten seconds. Finally, ten seconds were allowed for swallowing. After each slice, the participant was repeatedly asked to rate the pleasantness of the particular stimulus on a 21-point scale, anchored at both sides (−10 for very unpleasant to 10 for very pleasant). Each ten-second interval was marked with a non-disruptive sound and a relevant message appeared on the screen, prompting the subject to take the next step. Thus, each banana slice was consumed at an interval of 40 seconds, followed by a pause of approximately 15 seconds for rating.

Before proceeding with analysis, the data were closely inspected for outliers. The following stringent criteria were set to differentiate outliers from naturally occurring fluctuations: an observation that fell beyond two standard deviations from the group mean for each measure, and, at the same time, did so systematically, i.e. in at least 5 measures out of 10 was considered an outlier. Furthermore, the decision to remove such observations from the analysis was further supported by unreliable ratings of self-assessed hunger in which, despite the instructions to refrain from food 2 hours prior to participation, a 10 was given. Even taking into account the subjectivity of the assessments, such reports do not seem credible, be they an indication of the fact that the participant had misunderstood the scale, was careless about his or her responses or that he or she had ignored the instructions not to eat. On these grounds, one case from either group has been excluded from the analysis.

A mixed-design ANOVA with repeated measures (denoted by m1–m10) as a within-subjects factor and group (anosmic subjects and controls) as a between-subjects factor was used. Since for post hoc analysis of small samples nonparametric tests are recommended, we applied Wilcoxon matched pairs signed-rank test (exact test procedure) with Bonferroni correction to follow up the findings. In addition, effect sizes (as denoted by *r*) were computed. Statistica 8.0 was used for all data analysis. All results are reported as significant at p<.05 unless stated otherwise.

## Results

**Figure 1:**
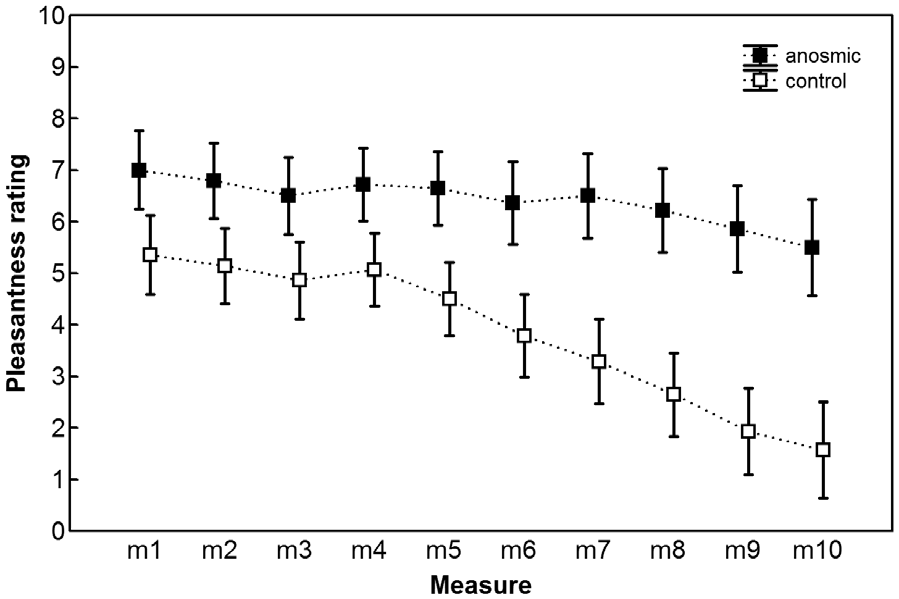
Pleasantness ratings. Pleasantness ratings (mean ± SE) across repeated measures (only the positive side of the scale is displayed).

The analysis yielded a significant main effect of group (F(1, 26) = 6.71, p = .02). Visual inspection of the data (see Fig. 1) suggests that anosmic participants consistently rated the stimuli as more pleasant than the control group.

Furthermore, there was a significant main effect of repeated measure (portion) upon pleasantness ratings (F(1.85, 48) = 12.15, p<.001). Mauchly's test indicated that the assumption of sphericity had been violated, χ^2^(44) = 268.07, p<.001, therefore degrees of freedom were corrected using Greenhouse-Geisser estimates of sphericity (ε = .21). Repeated contrasts revealed that there was a significant change (decrease) between m2 and m3, and m5 and m6 (both *p*s = .005), m7 and m8, and m8 and m9.

More importantly, a significant portion*group interaction was found (F(1.85, 48), p = .04 = 3.54). This turned out to be due to the differential change in pleasantness ratings in the congenital anosmics and controls between m6 and m7 (p<.01).

To determine whether there was a continuous significant decline in pleasantness ratings as compared to the baseline in the individual groups and to ascertain at which time point it commenced, we employed the Wilcoxon matched pairs signed-rank test with Bonferroni correction. Whilst multiple comparisons to baseline yielded no significant results at the specified level of significance (α = .006) in the anosmic group, in the control group there was a statistically significant decrease in pleasantness ratings between m1 and m8 (T = 6, p = .005, r = .52), m1 and m9 (T = 4.5, p<.005, r = .57), and m1 and m10 (T = 1.5, p<.005, r = .58).

## Discussion

In the present study, congenitally anosmic individuals exhibited a more sustained positive response to the stimulus over the course of consumption (relative to baseline) compared with the control group. One line of reasoning, somewhat speculative though, is that the mechanisms underlying hedonic habituation (resulting from repeated prolonged exposure to a simple food and, by extension, possibly also sensory-specific satiation), might be impaired as a consequence of the absence of the sense of smell. Thus, congenitally anosmic participants might exhibit a less-pronounced decline in the hedonic valence of a food than healthy controls do. In other words, to use the original definition, they may not ‘get bored with taste’ as rapidly as individuals with an intact sense of smell. However, we argue that the hypothesized ‘boredom with taste’ [11] should be conceived of as ‘boredom with flavor’ instead, due to smell and taste being closely intertwined in healthy individuals [28]. It is people with this kind of olfactory impairment who are truly in the position to appreciate the sense of taste separately from olfaction; our results indicate that their appreciation of taste might not diminish as rapidly as that of flavor in healthy individuals. However, a recent study [29] showed that sensory-specific *satiety* does not appear to be affected by olfactory dysfunction, as it developed in normosmic and hyposmic/anosmic individuals alike.

An alternative explanation is that being forced to focus on foods with specific characteristics in order to derive some enjoyment from eating may result in considerably fewer choices. In other words, in a world of bland flavors, congenitally anosmic individuals may exhibit a more sustained positive response than healthy subjects would when presented with a food that possesses some redeeming qualities. One of these is sweet tastes, as evidenced by the finding that individuals who have lost olfaction, the most ‘sophisticated’ sense to enjoy foods simply eat more sweet dishes to reward themselves [21]. Add to this the fact that there is evidence for a biologically-driven hedonic bias in preference for sweet taste [30] and it seems understandable why congenitally anosmic individuals would want to derive enjoyment from this particular food characteristic. Food texture might have been another candidate. Clearly, further studies employing a wide selection of diverse foods are needed to test this hypothesis.

Yet another possible explanation is that, being deprived of the sense of smell, which, to a variable extent, constitutes our experience of satiation [17], individuals with this type of olfactory disorder have to ‘make do with what they have left’. The knowledge that ten banana bites are usually not enough to ward off hunger, coupled with the limited array of dietary choices congenital anosmics find enjoyable, might have resulted in these participants experiencing a prolonged appreciation of the stimuli. Of course, however, this remains an idea for further research.

Finally, not only did the stimulus elicit a more sustained positive response in the congenitally anosmic participants but it was also rated as more pleasant. This might seem to contradict congenitally anosmic individuals' self-reports of reduced food appreciation in general (i.e. the longer-term overall degree of enjoyment); however, the aim of this study was to investigate the pattern of actual, immediate changes in appreciation of one particular simple, single-food meal over the course of consumption. This particular food may well have happened to be one of their “remedy” or “comfort” foods. Besides, the length of time for which the olfactory loss has been noted (along with the individual's age) appear to be important factors, as older subjects who had been aware of their olfactory loss for more than three years tended to indicate decreased food enjoyment less frequently than younger ones [22].

It is also crucial to understand that the ratings in both the congenitally anosmic and control group were assigned relative to other foods with which they had had experience throughout their lives. When the sense of smell is absent, not only will the pleasantness of food stimuli be judged on the basis of the remaining available sensory attributes, but it will also be judged in the context of non-olfactory experience. However, these interpretations of the general level of food appreciation must be treated with caution, as no non-olfactory stimuli to normalise the scale to have been employed in this study. Furthermore, only one particular stimulus was used in this study. Foods with different characteristics and palatability should be employed in future investigations to ascertain whether the present finding might generalize to other types of stimuli as well.

Although the nature of the present study is explorative, its findings point in the direction of the idea that, at least to some degree, congenital anosmia might affect the hedonic valence of a simple food and/or interfere with the development of sensory-specific satiation (or expression thereof). However, whether this is due to the absence of olfactory stimuli in congenital anosmia or an effect of other properties of this particular olfactory disorder, which have not been addressed in this study, remains to be further explored. The present explorative study contributes towards an issue deserving of more attention than it has been given so far and further investigations should be carried out to explore the possible role of olfaction in inducing or increasing perceived satiation, which, in turn, might lead to a decrease in food intake.
